# Conduction System Pacing Today and Tomorrow

**DOI:** 10.3390/jcm11247258

**Published:** 2022-12-07

**Authors:** Andreas Haeberlin, Siro Canello, Andreas Kummer, Jens Seiler, Samuel H. Baldinger, Antonio Madaffari, Gregor Thalmann, Adrian Ryser, Christoph Gräni, Hildegard Tanner, Laurent Roten, Tobias Reichlin, Fabian Noti

**Affiliations:** 1 Department of Cardiology, Inselspital, Bern University Hospital, University of Bern, 3010 Bern, Switzerland; 2 ARTORG Center for Biomedical Engineering Research, University of Bern, 3008 Bern, Switzerland; 3 Swiss Institute for Translational and Entrepreneurial Medicine, 3010 Bern, Switzerland

**Keywords:** conduction system pacing, left bundle branch area pacing, left bundle branch pacing, leadless pacing

## Abstract

Conduction system pacing (CSP) encompassing His bundle (HBP) and left bundle branch area pacing (LBBAP) is gaining increasing attention in the electrophysiology community. These relatively novel physiological pacing modalities have the potential to outperform conventional pacing approaches with respect to clinical endpoints, although data are currently still limited. While HBP represents the most physiological form of cardiac stimulation, success rates, bundle branch correction, and electrical lead performance over time remain a concern. LBBAP systems may overcome these limitations. In this review article, we provide a comprehensive overview of the current evidence, implantation technique, device programming, and follow-up considerations concerning CSP systems. Moreover, we discuss ongoing technical developments and future perspectives of CSP.

## 1. Introduction

Implantable cardiac pacemakers (PMs) have been the mainstay for the treatment of bradyarrhythmia for decades. While these devices undoubtedly represent a unique success story since their first implantation in 1958, PMs have evolved significantly during the past years. With the introduction of leadless devices and conduction system pacing, the major limitations of conventional systems such as transvenous leads or unphysiological myocardial stimulation have been addressed. Conduction system pacing systems in particular have introduced a large variety of pacing options, which allow for tailoring antibradycardia stimulation individually to the patient’s needs. On the other hand, implantation complexity has increased considerably, and novel follow-up challenges have emerged. The goal of this review is to provide a state-of-the-art overview of conduction system pacing techniques, specific challenges, and clinically important implantation and follow-up tips.

## 2. Conduction System Pacing–Concept and Evidence

Conduction system pacing (CSP) encompasses His bundle pacing (HBP) and left bundle branch area pacing (LBBAP). The general rationale of CSP is to maximally benefit from the heart’s intrinsic conduction system to stimulate the myocardium in the most physiologic and efficient way possible.

HBP was already performed more than 20 years ago in humans [1]. Shortly thereafter, this solution was followed by early attempts of left ventricular septal pacing using a dedicated novel lead design [2]. A refined approach for left ventricular bundle branch pacing was proposed by Huang et al. in 2017 [3]. Given the relatively recent introduction of these concepts, the available evidence for CSP is still limited compared to the exhaustive evidence underlining the benefits of cardiac resynchronization therapy (CRT), which, similar to CSP, aims at improving synchronous left ventricular activation. Subsequently, we provide a short overview of the key studies published so far that have investigated the use of HBP and LBBAP systems.

### 2.1. His Bundle Pacing

Soon after the first HBP system implantations, acute beneficial hemodynamic and echocardiographic effects compared to conventional right ventricular (RV) pacing were demonstrated. Ventricular activation time was shortened during selective and non-selective HBP compared to RV pacing [4]. HBP also decreased interventricular dyssynchrony and reduced mitral valve regurgitation [5]. After the implantation of HBP in lieu of CRT, tricuspid valve regurgitation was reduced as well [6]. These mechanistic effects seem to translate into clinical outcomes: In nonrandomized registry studies, HBP was associated with a reduction of the combined endpoint of mortality and heart failure hospitalizations during long-term follow-up compared to RV pacing [7,8].

In CRT candidates, HBP leads to an improved NYHA functional class by QRS shortening and improvement of the LV ejection fraction [9]. Two randomized trials (His-SYNC and His-Alternative) have also compared HBP to CRT in an on-treatment fashion. HBP was equivalent or even superior to CRT with respect to QRS narrowing compared to baseline and echocardiographic response rates [10,11]. However, a crossover was required in up to almost half of the patients in the HBP group due to procedural failure. In a very recent randomized crossover analysis, HBP provided a significant improvement in the LV ejection fraction compared to CRT after AV nodal ablation [12]. These studies strongly support the only class IIa recommendation for CSP in the latest European pacing guidelines: In case coronary sinus lead implantation is unsuccessful, HBP lead implantation should be considered as an alternative [13].

HBP is the most physiologic pacing strategy, although the technique suffers from major limitations. The significant learning curve during the first 50–100 lead implantations needs to be overcome first [14]. Reported success rates vary widely from 50%-92% depending on the indication and operator experience [9,15]. Success rates in the presence of bundle branch blocks or a complete AV block may hardly be higher than 50% even in experienced centers [15]. Furthermore, even after successful implantation, power consumption of the device is higher compared to conventional RV pacing and may further increase over time [7]. This shortens the battery life and may even require HBP lead revision or lead deactivation during follow-up.

### 2.2. Left Bundle Branch Area Pacing

LBBAP comprises true left bundle branch pacing (LBBP), left bundle fascicular pacing (LBFP), and left ventricular septal pacing (LVSP). Definitions and distinctions between these modalities, however, are not uniformly used and differ amongst studies. Therefore, caution is warranted when comparing different trials.

**Figure 1:**
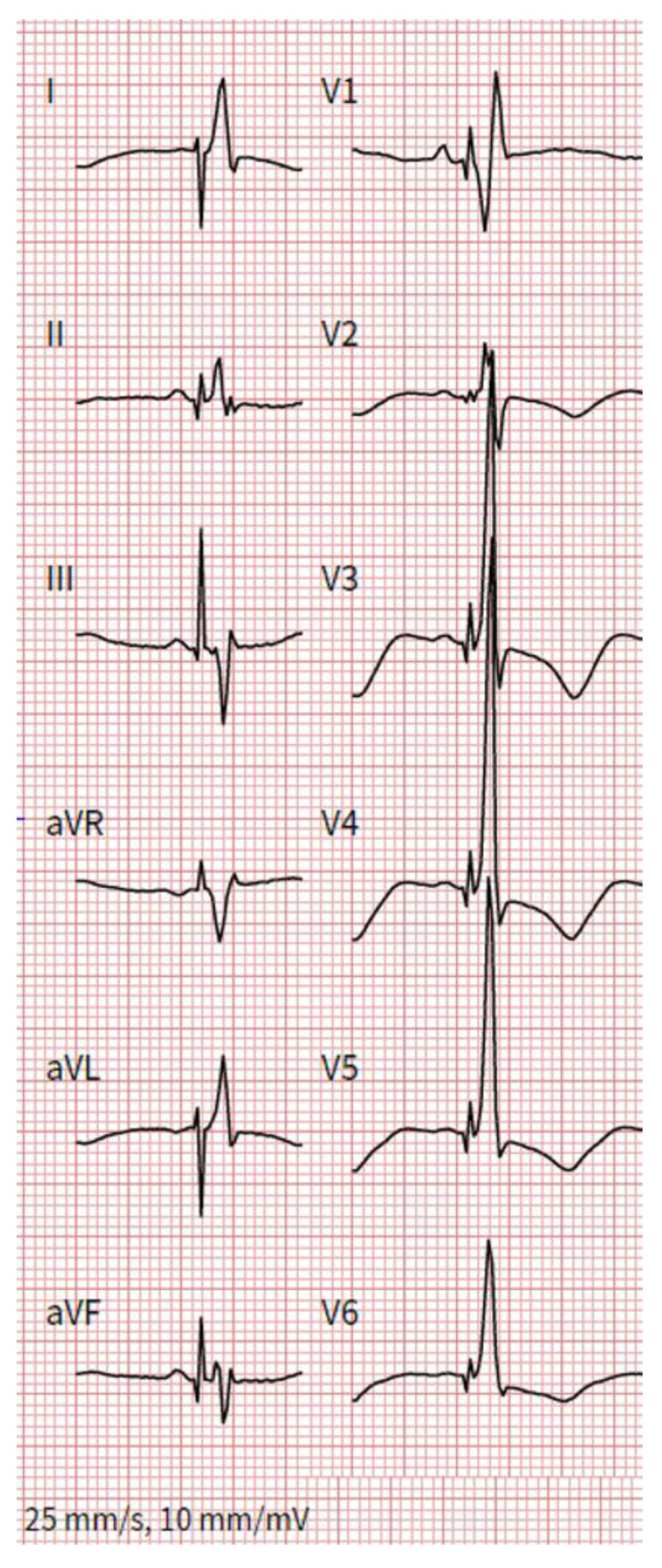
Paced QRS morphology in a patient with a dual-chamber PM and an LBBAP lead. V1 exhibits an R’, R-wave peak time in V6 is ~70 ms, the V1–V6 interpeak interval is ~35 ms and paced QRS duration is ~120 ms.

Compared to HBP, data on LBBAP are even more scarce and largely based on (smaller) registry studies. In general, capture thresholds are low and remain stable over time, and significant tricuspid valve regurgitation is rare [16]. Patients with a wide QRS complex benefit from the improvement of the ejection fraction [16]. LBBAP shortens the QRS complex significantly compared to RV pacing [17]. Clinically, this results in lower rates of heart failure hospitalization and even lower all-cause mortality according to registry data [17]. Despite pacing from a more distal site than HBP, the paced QRS duration of LBBAP is not significantly longer and—depending on the indication—in the range of 120 ms (Figure 1) [18]. Full correction of a left bundle branch block can more often and more easily be achieved compared to HBP.

Patients with a conventional CRT indication may benefit from LBBAP as well. In a large observational study that included 477 CRT and CSP patients (219 CRT, 171 LBBAP, and 87 HBP systems), the paced QRS duration was shorter in LBBAP systems (133 vs. 153 ms). Echocardiographic response rates were greater in patients receiving a CSP system compared to patients with conventional CRT. In patients with LBBAP, LVEF improved from 26 to 41% compared to 26 to 34% in the conventional CRT group [19]. This also translates into favorable clinical outcomes: The risk of death or heart failure hospitalization was reduced significantly. In a small propensity-score-matched analysis, LBBAP outperformed conventional CRT with respect to the functional NYHA class [20]. These promising findings are supported by the prospective randomized LBBP-RESYNC trial that included 40 patients with non-ischemic cardiomyopathy, a left bundle branch block, and CRT indication. LBBAP demonstrated greater improvement in the LV ejection fraction compared to conventional CRT even in the intention-to-treat analysis [21]. LBBAP is also promising in patients with right bundle branch block, heart failure, and mildly reduced LV dysfunction as it narrows QRS duration and improves LV function [22]. Thus, at least in the case of failed coronary sinus lead implantation in CRT candidates, LBBAP may—similarly to HBP—serve as a bail out with consecutive improvement of the LV ejection fraction (~10% increase compared to baseline) [23]. In an international multicenter study, LBBAP was performed after failed coronary sinus lead implantation and resulted in a QRS narrowing of almost 30 ms [23]. Unlike HBP, LBBAP offers excellent capture thresholds and R-wave amplitudes in this situation [23]. This makes LBBAP leads suitable for sensing in implantable cardioverter-defibrillators (ICDs), as was demonstrated in the CROSS-LEFT pilot study [24]. However, despite the larger target area for lead implantation, the challenges and learning curve of transseptal LBBAP lead implantation should not be underestimated. Up to 150 lead implantations are required to achieve optimal implantation results. The implantation duration and fluoroscopy time still remain slightly longer compared to conventional RV lead implantation [25]. Finally, a lead position deep in the interventricular septum raises concerns regarding the long-term extractability of LBBAP leads [26].

Success rates of LBBAP lead implantation attempts are >90% for conventional bradycardia indications and >80% for heart failure indications according to a European multicenter registry (MELOS) [27]. However, true full LBBP capture (not including fascicular pacing or LV septal pacing) may be achieved in <50% of cases [27,28]. Reported complication rates are approximately 8%, whereof 4% represent intraoperative perforations into the LV, which usually do not result in sequela after the intervention [27]. Since LBBAP is a very young technique, the latest European and American pacing guidelines refrained from any recommendations regarding its use at this point in time.

## 3. CSP Implantation Technique

CSP lead implantation differs from traditional lead implantation regarding several key aspects and is more challenging. High-resolution fluoroscopy, a 12-lead ECG, dedicated implantation material, and trained implanters are required for CSP lead implantation. The introduction of three-dimensional (3D) guiding sheaths allows for better reach of the target area and has boosted interest in CSP in the past few years.

### 3.1. Implantation Guiding Sheaths

Dedicated 3D guiding sheaths for CSP are available in various dimensions from Medtronic (Dublin, Ireland), Biotronik (Berlin, Germany), Boston Scientific (Marlborough, MA, USA), and Abbott (Chicago, IL, USA). They all require a 7–9F introducer sheath and provide a side-port for contrast delivery to perform septography. The sheaths facilitate reaching a suitable septal target site and provide additional backup, which may be of particular importance for LBBAP lead implantation, as the lead needs to penetrate deep into the interventricular septum. Some sheaths are deflectable or even equipped with mapping electrodes, which may be helpful for mapping the His bundle. The guiding sheaths are inserted using a J-wire at the beginning of the implantation and are slit at the end of the procedure.

### 3.2. CSP Leads

A lumen-less lead (3830 SelectSecure, Medtronic) with a 4.1F lead body and a 1.8 mm long fixed helix has initially been used for HBP in particular. However, for LBBAP, conventional stylet-driven leads (Solia S, Biotronik; Fineline II and Ingevity+, Boston Scientific; Tendril 2088TC, Abbott) have been used for CSP as well [29,30]. The lead implantation techniques vary slightly amongst the different electrode designs [29], and learning curves for the individual leads must be assumed. No systematic performance differences between lumen-less and stylet-driven leads regarding acute implantation success rates, screw-in attempts, and fluoroscopy time have been identified so far [30]. However, concerns regarding a higher rate of lead failures of extendable helix leads compared to fixed-helix leads have been raised recently [26].

### 3.3. Additional Material

Sterile cables with crocodile clamps allow for connecting the CSP lead to the electrophysiology system or a pacing system analyzer (PSA). This is essential for (pace) mapping, repetitive impedance measurements, and standard lead measurements.

### 3.4. HBP Lead Implantation Technique

The target site for HBP is fibers of the specific conduction system in the right atrium and the penetrating bundle, which is approximately 2–8 mm long and 1 mm thick. The HBP lead is advanced through the guiding sheath while mapping the target area in a unipolar fashion until a His bundle potential can be recorded. The lead is fixated by (usually) 4–8 clockwise turns of the whole lead. Selective or non-selective pacing (with correction of a left bundle branch block in the case of HBP in lieu of CRT) is ensured by uni- and bipolar pacing from the HBP lead. If correction of the QRS cannot be achieved, a His-optimized CRT (HOT-CRT) system with the additional implantation of a CS lead may be a more effective option, resulting in complex programming of the device. Distinguishing non-selective HBP from RV septal pacing may be challenging, thus, programmed stimulation and cutoff values for QRS duration, R-wave peak time in V6, and morphological criteria may help to identify the true His bundle capture [31,32]. Pacing thresholds should be <2.5 V@1 ms in non-PM-dependent patients and lower in dependent patients [33] (<1.5 V@1 ms in the author’s opinion). The implantation of a permanent RV backup lead is recommended in patients undergoing a pace-and-ablate strategy due to a potential threshold rise after subsequent AV nodal ablation [13] or PM-dependent patients due to the risk of dislocation of HBP leads.

### 3.5. LBBAP Lead Implantation Technique

**Figure 2:**
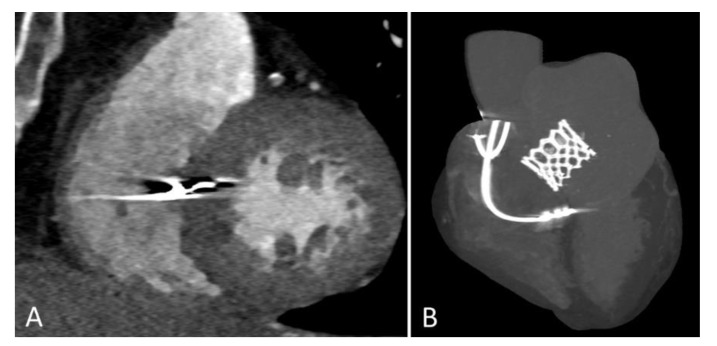
Computed tomography (CT) images of a patient with AV block after transcatheter aortic valve replacement and consecutive dual-chamber PM implantation with a ventricular lead in LBBAP position. (**A**) The deep septal implantation site of the CSP lead in a short-axis projection. (**B**) The basal implantation site in the proximal left bundle in a reconstruction. The resulting ECG is shown in Figure 1.

**Figure 3:**
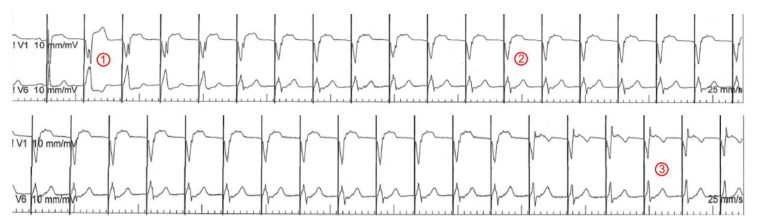
ECG leads V1 and V6 during LBBAP lead implantation. Continuous unipolar pacing is performed from the lead’s tip. ① A wide QRS reflecting right ventricular septal pacing with a notch near the nadir of the QRS (one of the criteria for a suitable starting position for lead drilling). ② During the drilling process, this notch starts to move towards the end of the QRS in V1 and the QRS duration is shortened (deep septal pacing). ③ Finally, an r’ appears in V1 and the QRS suddenly shortens again significantly, indicating CSP capture.

**Figure 4:**
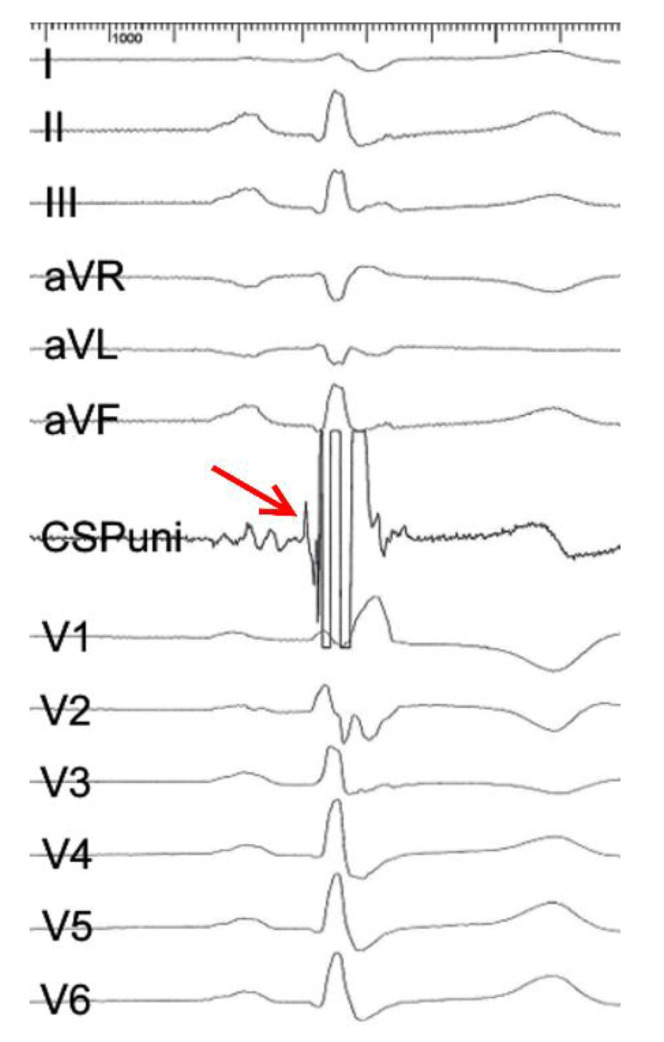
12-lead ECG from a patient, who underwent LBBAP lead implantation. The unipolar electrogram (CSPuni) shows a left bundle branch potential during intrinsic rhythm, which is indicative of successful positioning of the lead in the specific conduction system (arrow).

The target implantation area for an LBBAP lead is significantly larger [34] but the lead needs to be advanced through the interventricular septum until it reaches the LV subendocardium [35]. To identify a suitable starting point for the lead-drilling process on the right ventricular septal side, fluoroscopy, and pace mapping are used [36]. Contrast-enhanced imaging strategies may further facilitate lead implantation [37]. Subsequently, the lead is forced gradually through the septum by clockwise turns (Figure 2). Lead implanters should be aware of fixation issues such as lead entanglement (prevents septal penetration and destroys the lead) and drilling (favors lead dislocation). During the screw-in of the lead, continuous unipolar pacing is performed to monitor the fixation progress through the septum by recognizing ECG changes (Figure 3). Unipolar impedances <420Ω and the loss of injury current are specific indicators for undesired septal perforation [38]. Sensing and pacing thresholds are usually similar or even better compared with conventional RV lead implantation [27]. The 12-lead surface ECG is essential for the verification of conduction system recruitment. A Qr or qR complex in V1 is desired. Moreover, an R-wave peak time in V6 < 80 ms [39], a V1–V6 interpeak interval > 40 ms [40] (Figure 1), and a left bundle branch potential during an intrinsic rhythm (Figure 4) are highly specific for successful implantation of the lead in the left bundle.

## 4. Considerations on Device Programming and Follow-Up

CSP device programming and follow-up are challenging for several reasons. Up to now, there have been no designated devices in the market addressing CSP programming requirements. The CSP lead can be connected to the atrial, RV, or LV ports of the PM, which increases the overall complexity of CSP programming considerably. In addition, device follow-ups are more time-consuming and require a 12-lead ECG for threshold testing. The CSP lead may pace multiple structures simultaneously (e.g., conduction system with or without correction of a bundle branch block as well as myocardium). The proper identification of the individual thresholds of these structures is crucial for tailored programming of CSP systems [41].

HBP systems may suffer from poor CSP lead performance (low-amplitude ventricular signals, oversensing of atrial and His signals, and high thresholds), which makes programming His bundle PMs often cumbersome and challenging [42]. Moreover, non-selective and selective stimulation with or without correction of a bundle branch block needs to be distinguished from RV septal pacing, and the pacing output needs to be adjusted accordingly. Full correction of bundle branch blocks is desired if an HBP system is implanted in lieu of a CRT.

In contrast, programming LBBAP systems is relatively simple. The LBBAP lead is usually the only ventricular lead since a backup lead is rarely used and an additional coronary sinus lead does not provide better LV activation in the case of true LBB stimulation. For resynchronization, as in HOT-CRT, a CS-lead may be added if full recruitment of the left bundle cannot be achieved, resulting in a left-optimized CRT (LOT-CRT).

In Table 1, we provide an overview of the most important programming recommendations for LBBAP systems with a CSP lead in a ventricular port of the device.

## 5. Future Perspectives

### 5.1. Clinical Evidence

Currently, published studies investigating CSP are often monocentric, retrospective, and descriptive in nature. Randomized studies investigating CSP approaches are scarce, but numerous trials have been initiated. Amongst these, only some studies are briefly introduced here. The multicentric PROTECT-HF study aims to randomize 2600 patients with bradycardia pacing indication and a mildly reduced LV ejection fraction to either RV pacing or CSP (HBP or LBBAP) with the primary composite endpoint of death and heart failure hospitalizations. The smaller LEAP-BLOCK study is a randomized controlled trial designed to investigate whether LBBAP reduces the risk of RV-pacing-induced cardiac dysfunction compared to conventional pacing in patients with an AV block and normal LV function.

Randomized trials comparing CSP and CRT systems have also been initiated but encompass only slightly more than 100 patients each. The CONSYST-CRT trial compares both modalities in patients with a class I or IIa CRT indication in a randomized non-inferiority fashion. Resynchronization will be provided by a conventional coronary sinus or a CSP lead. A composite endpoint of mortality, cardiac transplantation, heart failure hospitalization, and insignificant improvement of the LV ejection fraction after implantation will be analyzed. The LEFT-BUNDLE-CRT trial is a similar analysis with 176 patients qualifying for CRT-P/CRT-D implantation. The primary non-inferiority outcome measure will be a positive response to resynchronization defined by a clinical score or LV end-systolic volume.

While all these studies will be important to assess the future prospects of CSP, key limitations have to be considered at this early stage of CSP system evolution. The limited implantation success rate and the long learning curve for implanters will inevitably result in a significant amount of crossovers in randomized trials hampering intention-to-treat analyses. Moreover, non-uniform success criteria used for LBBAP will impair comparability amongst studies.

### 5.2. Technical Improvements

The introduction of dedicated delivery systems for CSP leads has enabled operators to routinely implant CSP leads. However, the available systems are still far from perfect, and manufacturers are constantly improving CSP hardware.

Delivery sheaths have improved significantly with the introduction of different lengths, curves, and braided tubes for variable stiffness along the sheath body. Nonetheless, the manufacturer’s sheath portfolios still lack valuable tools (e.g., a longer sheath for delivery of stylet-driven leads for larger patients).

Future PM generators will offer the possibility to select the conduction system as a pacing site. Nominal programming values will be suggested accordingly, facilitating device programming. Moreover, improved dedicated CSP lead designs are under development. Lead prototypes include various designs such as fixed-screw leads offering a longer screw and a tempered tip design for better septal penetration.

**Figure 5:**
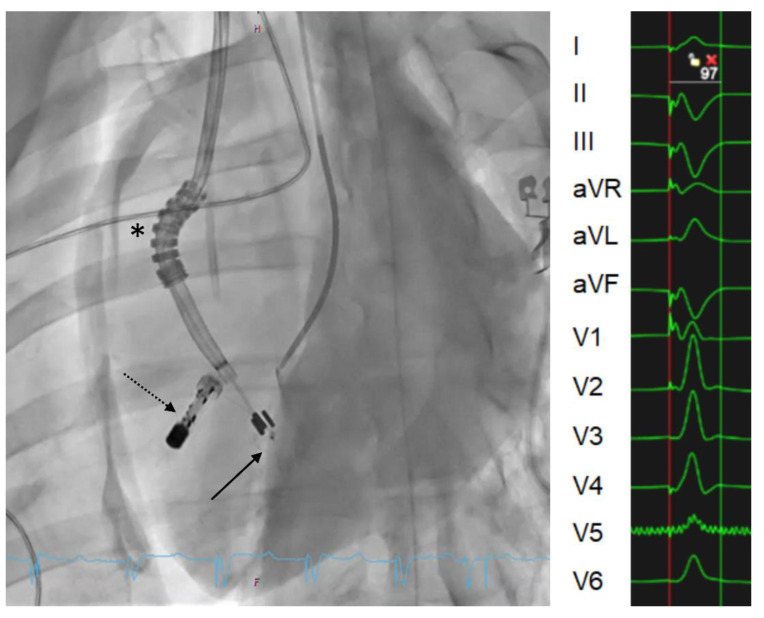
Implantation of a leadless CSP PM in a domestic pig. The steerable implantation catheter (asterisk) allows positioning the stimulation electrode towards the interventricular septum (solid arrow). The main body of the leadless PM is implanted in an apical position (dotted arrow). The corresponding ECG with a simulated QRS duration of <100 ms is shown on the right.

Finally, merging leadless technology and conduction system pacing approaches may be a highly desirable form of pacing in the future. Leadless systems have evolved rapidly during the past years and novel VDD systems provide relatively reliable AV synchrony [43]. True dual-chamber systems using conductive communication [44] are undergoing first-in-man trials. The stimulation electrode of the ventricular device in such systems might be positioned in the conduction system via a transseptal route. Such PMs may omit pocket and important lead-related complications while delivering a more physiological form of pacing than contemporary leadless PMs. A prototype of a leadless CSP PM undergoing pre-clinical in vivo implantation is shown in Figure 5.

## 6. Conclusions

Conduction system pacing is becoming increasingly popular. Novel hardware solutions provided by manufacturers and dedicated training courses for implanters facilitate the adoption of this new technique. However, despite the general enthusiasm CSP faces nowadays, structured evidence from randomized trials is still limited. Data are very encouraging but whether or not CSP will be the new Holy Grail for antibradycardia pacing remains to be investigated further. Thus, despite an increasingly liberal implantation practice, patient-specific counseling and case-by-case decisions seem advisable. 

## Figures and Tables

**Table 1:** LBBAP programming recommendations for systems with a CSP lead in a ventricular port.

Parameter	Recommendation	Reasoning
Sensing vector	Bipolar	Bipolar sensing is preferred to avoid oversensing of pectoral myopotentials.
Sensitivity	As a conventional lead	Sensing values are usually excellent and rarely cause concerns.
Output polarity	Bipolar (extended bipolar/unipolar may be considered for better visibility of the PM stimulus)	Bipolar pacing is usually more power-efficient but simultaneous cathodal and anodal capture may mimic lead microdislocation. Be aware of undesired anodal RV capture in case of an extended bipolar configuration.
Output voltage	As a conventional lead (2× threshold, 0.4 ms pulse duration)	Capture thresholds are usually excellent and remain stable over time.
AV delay	LBBAP lead in RV port: subtraction of LBB-V interval from desired AV delay LBBAP lead in LV port: program VV delay first, adjust AV delay thereafter in a similar fashion	The resulting AV delay corresponds to the programmed AV delay plus the conduction time from the left bundle to the ventricle (LBB-V interval, ~15–25 ms).
VV delay	LBBAP lead in LV port: option 1: program LV first (~60–80 ms, in case of LV septal pacing, fusion may shorten QRS) option 2: deactivate RV pacing in non PM-dependent patients option 3: program subthreshold stimulation in the RV	Promote ventricular activation via the conduction system in case of proper left bundle activation. In case of deep septal lead position, LV-RV-fusion (similar to CRT) may further decrease QRS duration. Subthreshold stimulation may cause confusion and provides no ventricular backup.
Automatic capture control	Monitor	May be activated in the chronic stage when correct functioning has been verified.
Automatic sensing	On	Sensing values are usually excellent and rarely cause concerns.
Ventricular safety pacing	On	Turn off if CSP lead is connected to the atrial port (after crosstalk test).

## Data Availability

Not applicable.
